# Artificial intelligence as a medical device in radiology: ethical and regulatory issues in Europe and the United States

**DOI:** 10.1007/s13244-018-0645-y

**Published:** 2018-08-15

**Authors:** Filippo Pesapane, Caterina Volonté, Marina Codari, Francesco Sardanelli

**Affiliations:** 1 Postgraduation School in Radiodiagnostics, Università degli Studi di Milano, Via Festa del Perdono 7, 20122 Milan, Italy; 2 Independent Researcher, 3 Greenwich Court, Cavell Street, London, E1 2BS UK; 3 Unit of Radiology, IRCCS Policlinico San Donato, Via Morandi 30, 20097 San Donato Milanese, Milan, Italy; 4 Department of Biomedical Sciences for Health, Università degli Studi di Milano, Via Morandi 30, 20097 San Donato Milanese, Milan, Italy

**Keywords:** Artificial intelligence, Legislation, Policy, Privacy, Radiology

## Abstract

**Abstract:**

Worldwide interest in artificial intelligence (AI) applications is growing rapidly. In medicine, devices based on machine/deep learning have proliferated, especially for image analysis, presaging new significant challenges for the utility of AI in healthcare. This inevitably raises numerous legal and ethical questions. In this paper we analyse the state of AI regulation in the context of *medical device* development, and strategies to make AI applications safe and useful in the future. We analyse the legal framework regulating medical devices and data protection in Europe and in the United States, assessing developments that are currently taking place. The European Union (EU) is reforming these fields with new legislation (General Data Protection Regulation [GDPR], Cybersecurity Directive, Medical Devices Regulation, In Vitro Diagnostic Medical Device Regulation). This reform is gradual, but it has now made its first impact, with the GDPR and the Cybersecurity Directive having taken effect in May, 2018. As regards the United States (U.S.), the regulatory scene is predominantly controlled by the Food and Drug Administration. This paper considers issues of accountability, both legal and ethical. The processes of medical device decision-making are largely unpredictable, therefore holding the creators accountable for it clearly raises concerns. There is a lot that can be done in order to regulate AI applications. If this is done properly and timely, the potentiality of AI based technology, in radiology as well as in other fields, will be invaluable.

**Teaching Points:**

• *AI applications are medical devices supporting detection/diagnosis, work-flow, cost-effectiveness.*

• *Regulations for safety, privacy protection, and ethical use of sensitive information are needed.*

• *EU and U.S. have different approaches for approving and regulating new medical devices.*

• *EU laws consider cyberattacks, incidents (notification and minimisation), and service continuity.*

• *U.S. laws ask for opt-in data processing and use as well as for clear consumer consent.*

## Introduction

*Artificial intelligence* (AI) is a branch of computer science dedicated to the creation of systems that perform tasks that usually require human intelligence, with different technical approaches [1]. The term AI is used to describe computer systems that mimic cognitive functions, such as learning and problem-solving [2]. These systems are currently based on artificial neural networks, which are flexible mathematical models using multiple algorithms to identify complex nonlinear relationships within large datasets [3], nowadays known as *big data.* Huge amounts of information can be retrieved from electronic archives, which could hardly be analysed, searched, or interpreted using traditional data-processing methods. Big data includes data from mobile phone applications, wearable technology, social media, environmental and lifestyle-related factors, socio-demographics, *omic* data (e.g., genomics, metabolomics, proteomics, radiomics), and data from standardised electronic health records or precision medicine platforms [4].

Among the health data, medical images such as those obtained through x-ray, computed tomography (CT), magnetic resonance imaging (MRI) and ultrasound examinations constitute one of the most interesting types of data, with high potential for research and clinical applications. Among other things, these data could help in improving the automatic detection of diseases (while minimising human errors) [5], creating study protocols [6], improving image quality and decreasing radiation dose [7], decreasing MRI scanner time [8], optimising staffing and scanner utilisation, thereby reducing costs [9], and offering the possibility of performing expensive and time-consuming screening programs in countries that otherwise cannot afford them [10, 11].

*Machine learning* is a term introduced by Arthur Samuel in 1959 [12] to define a field of AI where computers learn automatically from data accumulation; it has been extensively applied for big data analysis [13]. Within the machine learning domain, *deep learning* has emerged as a highly promising approach for image processing [14]. Unlike software, which requires specific instructions to complete a task, deep learning allows the system to recognise patterns independently and make predictions [15].

The deep learning modelling of big data exerts major influences on modern society, from web searching to financial technology banking, from facial recognition to medical decision support [16, 17]. The application of AI will change the working methodologies of many professionals, including physicians, and this will happen in radiology more quickly than in other medical fields.

Radiologists, having been pioneers of the digital era in medicine, can now accept AI as a new partner in their profession, along with a potential for a higher role of radiology in healthcare, as we have shown in a previous article [18]. However, there are challenges to AI applications in medicine and specifically in radiology that depend not on physicians, but on regulatory institutions and governments [19]. In this paper we analyse the issues related to recent policy initiatives to regulate AI in the context of *medical device* development, the accountability of AI under the law, and the implications of data protection and cybersecurity. The main differences between the policy of the European Union (EU) and that of the United States (U.S.) will be considered.

## Challenges of AI in medicine and radiology

The advancement in algorithm development combined with the ease of accessing computational resources currently allows AI applications to be used in medical decision-making tasks with promising results [14, 17]. The utilization of AI techniques in radiology represents a relevant topic for research teams. Deep learning algorithms are currently used in mammography for breast cancer detection [20], in CT for colon cancer diagnosis [21], in chest radiographs for the detection of pulmonary nodules [22], in MRI for brain tumour segmentation [23] and for diagnosis of neurologic disorders, such as Alzheimer disease [24].

The widespread enthusiasm and dynamism regarding the development of software based on AI in radiology is shown by the highly positive trend of publications in the literature in the last 10 years: from 100 to 150 to 700–800 yearly [18].

However, some ethical challenges are straightforward and need to be guarded against. Notably, one of them is the concern that algorithms may mirror human biases in decision making. Since healthcare delivery already varies by ethnicity, it’s possible that some ethnical biases could inadvertently be built into medical algorithms. AI applications introduced in nonmedical fields have already been shown to make problematic decisions that reflect biases inherent in the data used to train them [25]. Recently, a program designed to aid judges in sentencing by predicting an offender’s risk of recidivism have shown an unnerving propensity for discrimination [26]. Similarly, an algorithm designed to predict outcomes from genetic findings may be biased if there are no genetic studies in certain populations [25].

The intent behind the design of AI also needs to be considered, because some devices can be programmed to perform in unethical ways. For example, Uber’s algorithm tool ‘Greyball’ was designed to predict which ride hailers might be undercover law-enforcement officers, thereby allowing the company to identify and circumvent local laws [25]. Also, Volkswagen’s algorithm allowed vehicles to pass emissions tests by reducing their nitrogen oxide emissions when they were being tested [25]. Analogously, private-sector designers who create AI algorithms for clinical use could be subject to similar temptations, programming AI systems to guide users toward clinical actions that would generate increased profits for their purchasers (such as by recommending drugs, tests, or medical devices in which they hold a stake or by altering referral patterns) but not necessarily reflect better care [25]. These examples show the urgency for serious regulations and policy initiatives about the use of AI, especially in complicated care practices in which the correct diagnosis of a disease and the best management of a patient can be controversial.

## Regulatory issues and policy initiatives

AI systems do more than process information and assist officials to make decisions of consequence. Many systems — such as the software that controls an airplane on autopilot or a fully driverless car — exert direct and physical control over objects in the human environment [27]. Other systems, including medical and radiological devices, provide sensitive services that, when performed by physicians, require training and certification [15, 19, 27–29]. These applications raise additional questions concerning the standards to which AI systems are held and the procedures and techniques available to ensure those standards are being met [30]. What about technology under development today, such as autonomous imaging readers, whose very value turns on bringing skills into an environment where no one has them? And how do we think about systems that purport to dispense health advice, which requires adherence to complex fiduciary and other duties pegged to human judgement [31]?

Since unambiguity is one of the pillars of any legislation [32], the first aspect policymakers need to address when regulating any new field is *definitions*. This aspect becomes an actual issue with AI, a term that itself contains ambiguities: *what is intelligence?* What human abilities are to be considered the milestones that the AI systems need to accomplish? The AI’s lack of a stable, unanimous definition or instantiation complicates efforts to develop an appropriate policy infrastructure. Moreover, we might question the utility of the word *policy* in describing societal efforts to channel AI in the public interest. There are other terms in circulation. For instance, *governance* instead of *policy*: a new initiative anchored by the Media Lab of the Massachsetts Institute of Technology and the Berkman Klein Center for Internet and Society of the Harvard University refers to itself as the ‘Ethics and Governance of Artificial Intelligence Fund’ [33]. Anyway, we need a policy for the governance of AI in medicine (and radiology).

The second issue for policymakers is whether *to consider AI software used in healthcare as a medical device for legislave purposes*. Both the EU and the U.S. have their own criteria for identifying healthcare and medical devices, although both definitions share a purpose-based approach.

We will consider below what medical devices are and how legislation differs in these two geopolitical areas. However, it is interesting to point out that not all AI programs used in healthcare will be deemed to be medical devices. For example, as Tsang et al. [34] pointed out, “programs that analyse large amounts of data to develop knowledge about a disease or condition, rather than to decide on treatment options for an individual patient, may not necessarily be considered as having a medical purpose, and hence as a medical device”. It is the basic distinction between those research programs that *enhance medical knowledge* from those that *promote changes in healthcare*. How will the latter systems be regulated?

According to Thierer et al. [35] there are two main approaches that policymakers can use when regulating AI systems. One is the *precautionary principle* approach, which imposes some limits or sometimes outright bans on certain applications due to their potential risks. This means that these applications are never tested because of what could happen in the worst-case scenarios. The other approach is the so-called *permissionless innovation* approach, which allows experimentation to proceed freely, and the issues that do arise are addressed as they emerge. In 2016, Scherer [36] distinguished, instead, between *ex ante* and *ex post* regulation. Ex ante regulation is pre-emptive and tries to foresee the risks, similarly to the precautionary principle [35], while ex post regulation is retrospective and focuses more on providing a remedy to harm that actually occurred [37], and it is similar to the permissionless innovation approach mentioned above [35].

On the one hand, ex post regulation is hindered by the autonomous nature of AI systems, which evolve and change constantly according to their experiences and learning, in an unforeseeable way [38]. On the other hand, ex ante regulation is obstructed by AI applications *discreetness* (their development requires little physical infrastructure), *discreteness* (their components can be designed by different subjects, for different purposes and without actual coordination), *diffuseness* (these subjects can be dispersed geographically and yet collaborate on the same project), and *opacity* (it can be difficult for outside observers to identify and understand all the features of an AI system) [36].

Finally, a further issue for policymakers is *time*. Nowadays, companies understand the potential of machine/deep learning and are continuously collecting new types of data to analyse and exploit [39]. In an environment like the technology world and AI, which changes quickly and unpredictably, regulations need to be timely to be relevant.

These premises explain why regulation of medical devices is so controversial and subject to the vagaries of guidelines and subjective interpretations by the authorities. We consider below the regulatory minefield and the circumstances in which a software is regulated as a medical device in the EU and in the U.S.

Generally, while in the U.S. AI the technology sector prospered in a permissionless innovation policy environment, in the EU decision-makers adopted a different policy for this revolutionary technological branch [35]. Certainly, swifter approval of AI medical devices helps generate revenue for manufacturers, and physicians may benefit from having more tools at their disposal. But the final goal of bringing new devices to market should be to improve prevention, diagnosis, treatment, prognosis of diseases with a potential positive impact on patient outcome. Therefore, systems for approving new medical devices must provide pathways to market for important innovations while also ensuring that patients are adequately protected. To achieve these goals, the EU and the U.S. use different approaches [40].

## The EU approach

In the EU, the definition of medical device is provided by Article 1(2) of Directive 93/42/EEC [41]: the term *medical device* is applied to any instrument or other tool, including any kind of software, intended by the manufacturer to be used for human beings for the purpose, among others, of diagnosis, prevention, monitoring, treatment, or alleviation of disease. This definition has been endorsed by the MEDDEVs, non-legally binding guidelines drafted by the European Commission to guide stakeholders in complying with legislation related to medical devices [42].

The European regulatory regime currently in force flows from three directives on medical devices [41, 43, 44] and it requires manufacturers to ensure that the devices they produce are fit for their intended purpose. This means that they must comply with a number of *essential requirements* set out by the directives themselves. Depending on the risk classification of the device, whether the essential requirements have been met can be assessed either by the manufacturer or by a notified body, which is an independent accredited certification organisation appointed by the competent authorities of EU Member States.

This regulatory framework has been reformed by the new Medical Devices Regulation (MDR) [45] and the new In Vitro Diagnostic Medical Device Regulation (IVDR) [46] (Table 1). Both came into force on 25 May 2017, however the MDR will apply from 26 May 2020 while the IVDR will apply from 26 May 2022. Because they are *regulations*, as opposed to *directives*, once they apply they do so directly, without the need for the governments of EU member states to pass legislation to implement their scope [47] (Table 2). This reform originated from the awareness that the existing directives, created in the 1990s [41, 43, 44], are not fit to deal with new, evolving technologies, including AI systems, and from the identification of some flaws of this regulatory system, for example lack of control on notified bodies.

**Table 1:** Regulatory framework in the EU on medical devices

Directive 93/42/EEC	Directive on medical devices Will be replaced by MDR on 26 May 2020
MEDDEVS	Non-binding guidelines on legislation related to medical devices
MDR	Regulation on medical devices Applies from 26 May 2020 Repeals Directive 93/42/EEC
IVDR	Regulation on in vitro diagnostic medical devices Applies from 26 May 2022

*MDR, Medical Device Regulation; IVDR,* In Vitro *Diagnostic Medical Device Regulation; EEC, European Economic Community*

**Table 2:** Main differences between Directives and Regulations

Directives	≠	Regulations
The directives set out the objectives that must be attained. Once they are in force, EU member states have a limited period of time to implement national legislation that will satisfy those objectives.		Regulations are applied directly in EU Member States, without the need for national legislation to implement their purposes. Once they are in force, member states must comply with them.

*EU* European Union

Some of the main characteristics of this reform will be: extended scope to include a wider range of products, extended liability in relation to defective products, strengthening of requirements for clinical data and traceability of the devices, more rigorous monitoring of notified bodies, and improved transparency through making information relating to medical devices available to the public [48].

## The U.S. approach

In the U.S., regulatory approval allowing machines to do the work of trained radiologists is a major obstacle still unsolved. The amount of testing and effort necessary to secure clearance from the U.S. Food and Drug Administration (FDA) for allowing machines to provide primary interpretations of imaging studies without a radiologist would be overwhelming.

At the end of 2016, the 21st Century Cures Act [49] clarified the scope of FDA regulatory jurisdiction over software used in healthcare, specifying that a medical device is an instrument or other tool “intended for use in the diagnosis of disease or other conditions, or in the cure, mitigation, treatment, or prevention of disease, in man or other animals, or intended to affect the structure or any function of the body of man or other animals” [50]. There are some other factors narrowing the definition, but this is the most important for the purposes of this paper.

Every AI system falling within this definition will be regulated by the FDA, as provided by the Federal Food, Drug and Cosmetic Act [34] (Table 3). The FDA categorises the medical devices into three classes, according to their uses and risks, and regulates them accordingly. The higher the risk, the stricter the control. Class III is the category which includes the devices involving the greatest risk.

**Table 3:**
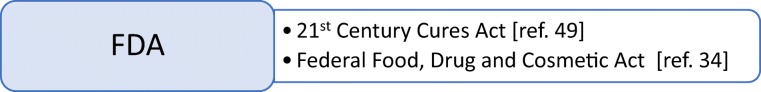
Regulatory framework in the USA on medical devices

*FDA* Food and Drug Administration

The *black box* nature and the rapid growth of machine/deep learning applications will make it difficult for the FDA to approve in a timely fashion all the new medical devices that are continuously being developed, given the volume and the complex nature of testing and verification involved. An example was the introduction of computer-assisted detection software for mammography in 1998 [51], which took many years and extensive lobbying to obtain clearance from the FDA to be used as a second screening reader [52]. Compared to computer-aided detection systems, FDA clearance is even harder to be obtained for an AI system that does not need radiologist’s supervision and cannot be compared to predicated medical devices used as replacements for radiologists. For this reason, developers nowadays present AI systems as aids tools for radiologists rather than as tools that substitutes for them [53]. This is not only a legal issue: it implies a relevant discussion about ethical responsibility, as we will see below.

## Data protection and cybersecurity implications

After the recent ferment about the Cambridge Analytica/Facebook scandal with personal data misuse [54], an ongoing debate about balance between privacy and better user experience (achieved via usage of personal data) is developing, especially when it comes to sensitive data such as medical information. The concept of circulating enormous amounts of confidential information in vast numbers of copies between many unregulated companies is increasingly insane and risky. Therefore, in the last decade personal data regulation is increasing and privacy concerns are growing [55].

However, we still need data as an integral part of technology development, especially for AI. As we discussed above, deep learning algorithms require a huge amount of data and powerful computers, and they usually take a long time to be trained because of the many parameters under consideration [19, 28]. The lack of well-annotated big datasets for training AI algorithms is a key obstacle to a large introduction of these systems in radiology [3, 13, 56]. Access to big data of medical images is needed to provide training material to AI devices, so that they can learn to recognise imaging abnormalities [13, 57]. One of the problems is that sensitive data might either be harvested illicitly or collected from unknown sources [58] because of the lack of unique and clear regulations [59].

In the era of electronic medical records, AI complicates an already complex cybersecurity landscape [60]: the concept of confidentiality requires that a physician withholds information from the medical record in order to keep it truly confidential [25]. Once a clinical decision based on AI is integrated into clinical care, withholding information from electronic records will become increasingly difficult, since *patients whose data are not recorded cannot benefit from AI analyses* [25]. *The implementation of deep learning systems will therefore require a reimagining of confidentiality and other core tenets of medical ethics.* Before using government over-regulation, we need to face the data protection and cybersecurity implications technologically. Data protection can no longer rely on current technologies that allow spreading of personal data and require data sharing at a large and uncontrolled scale [61].

In radiology, and in medicine in general, AI medical devices should use deep learning to provide data about patients without requiring their personally identifiable information in exchange. Currently, the use of AI raises two issues relating to the data collected by the devices. On the one hand, data must be protected from the same bodies collecting them. On the other hand, the same data are threatened by cyberattacks to these bodies as well as to the devices themselves.

## Data protection in the EU

Because of these threats, European regulators have decided to update the legislation concerning data protection and cybersecurity (Table 4). The General Data Protection Regulation (GDPR) applies from 24 May 2018, substituting the European legal framework for data protection as set out by Directive 95/46/EC [62]. According to the GDPR, all data processing and use should be opt-in, and consumer consent for data use should be clear. In general, *the GDPR completely prohibits current data marketing based on third-party non opt-in personal data*. The GDPR is a more suitable instrument to regulate AI because it has an extended territorial scope and wider rights for data subjects. For instance, enhanced notification requirements (under Article 33, personal data breaches must be notified to the supervising authority within 72 h), and rights to compensation for material or non-material damage and additional liability for data controllers and processors (Article 88):^1^ [63].

**Table 4:** Regulatory framework in the EU on data protection

Directive 95/46/EC	Directive on data protection Has been replaced by the GDPR
GDPR	Regulation on data protection Applies from 24 May 2018 Replaces Directive 95/46/EC
Directive (EU) 2016/1148	Directive on cybersecurity Applies from 10 May 2018

*EC, European Community; GDPR, General Data Protection Regulation; EU, European Union*

Again, because it is a regulation, the GDPR applies directly, and institutions have to comply with it starting from 24 May 2018 (which is why 2 years have been allowed for implementation since its enforcement on 24 May 2016).

In addition, the EU adopted the Cybersecurity Directive [64], which had to be implemented by the member states by 10 May 2018. This sets out a number of requirements for EU member states which aim to prevent cyberattacks and keep their consequences under control. Among other things, member states are required to ensure that operators of essential services take appropriate measures to prevent and minimise the impact of incidents and to preserve service continuity (Articles 14(2) and 16(2)), and to ensure that supervisory authorities are notified of incidents without undue delay (Articles 14(3) and 16(3)) [64].

## Data protection in the U.S.

While in the U.S. government regulation is less strict, cases like Cambridge Analytica/Facebook should remind the government that actions should be taken, and that the behaviour of companies needs changes.

The Health Insurance Portability and Accountability Act (HIPAA) is a compliance focus for health information concerns [34]. This act elaborates rules requiring, among other things, the formulation of policies and the setup of training systems for those who have access to sensitive data [34] (Table 5). Moreover, HIPAA does not hinder the action of individual states, where it protects further the individual’s right to privacy.

**Table 5:**
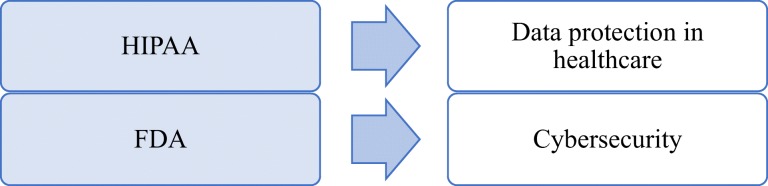
Regulatory framework in the USA on data protection

*HIPAA* Health Insurance Portability and Accountability Act, *FDA* Food and Drug Administration

Cybersecurity is dealt with by the FDA, which requires manufacturers to report only a limited number of risks their devices present and actions taken to minimise the vulnerability [34] (Table 5). The black box features of deep learning and the lack of transparency regarding how results are obtained have thorny legal implications [58]. In some cases, even the designers do not completely understand how AI algorithms process the data [58], so that *the lack of transparency is, basically, a lack of knowledge*. Considering the current amount of data collected, and that with an increased presence of AI applications this can only grow, regulatory actions regarding cybersecurity will face continuous challenges.

## Accountability and responsibility: an “internal evidence” from AI in the frame of evidence-based medicine?

Alongside the actual regulation and data protection, there are other legal implications of AI and its use, whether in healthcare or elsewhere. One of these is *accountability*. As soon as AI starts making autonomous decisions about diagnoses and treatments, moving beyond its role as merely a support tool, a problem arises as to whether its developer can be held accountable for its decisions. The first question is: who will be sued if an AI-based device makes a mistake?

Errors in AI appear mainly when confounding factors are correlated with pathologic entities in the training datasets rather than actual signs of disease. When AI devices decide, their decision is based on the collected data, the algorithms they are based on, and what they learnt since their creation. The reason their decisions are unpredictable is twofold [36]. On the one hand, *AI devices, even if they mimic human brain neural networks, think differently than humans*. Better, they think more quickly and accurately [38, 56, 58]. There is a huge number of possibilities in every given situation, and humans are unable to process all of these and consider them in order to make a decision. We consider only what is more obvious for our brains, while AI systems can consider every potential scenario and every consideration [2, 3, 15, 29, 39, 56, 65]. Because of this, *where faced with a decision to make, we do not share a common basis with AI devices*. *Therefore, we are unable to predict what they will decide in a given set of circumstances*. On the other hand, AI systems are designed to learn from their genuine experiences, and these are by their very nature unpredictable. Because it is not possible to foresee what experiences a system will encounter, neither is it possible to foresee how the system will develop. For these reasons, it is worth considering, when something ‘goes wrong’ following a decision made by an AI application, whether the device itself or its designer/builder is to be considered at fault. Will the designer be deemed negligent for not having foreseen what we have described as unforeseeable? Or for having left the possibility for development of the AI-device that would bring it to that decision?

AI will play an increasingly important part over the next years in the relationship between doctors and patients [15, 19, 35, 53, 66] and it will need to be bound by core ethical principles, such as beneficence and respect for patients, which have guided clinicians during the history of medicine [25]. We should remember, indeed, that *the radiologist, as a physician, is much more than simply an interpreter of images*. The duties of a practicing radiologist also include communication of findings, quality assurance, quality improvement, education, interventional radiology procedures, policy-making, and many more tasks that cannot be performed by computer programs [2]. The ability to provide a nuanced interpretation for complex findings, medical judgement, and wisdom of an experienced radiologist is difficult to quantify and even more difficult to simulate with AI systems [15].

Given the evolving complexity of AI technology, it is almost inevitable that some of its inner workings will appear to be a ‘black box’ to all but the very few who can comprehend how they work [58]. But that does not mean accountability is out of the question.

Importantly, in a broader theoretical framework, we should distinguish between *data analysis* (in this case, the AI-device output) and *decision making*. In the context of evidence-based medicine [67], the *best external evidence* (data from high-quality studies and meta-analysis, guidelines from governmental bodies and medical societies) has to be combined with *patients’ preferences and values* by using our *personal medical/radiological expertise*. We could say that, introducing AI in radiology, we have also a kind of *internal evidence* coming from the application of AI to the patients’ imaging procedure(s). One can argue that AI might, at least in the future, be able to do this job instead of physicians. However, when patients’ preferences and values play a non-negligible role (and hopefully this factor will not reduce its weight in the future), *human interaction and empathy by a physician/radiologist and a patient remain a fundamental dimension*. Indeed, AI will allow radiologists to have more time to communicate with patients and to confer with colleagues in multidisciplinary teams, as they will be less busy doing routine and monotonous tasks that can be effectively performed by computers [53].

Accountability for an AI output may be also a simple matter for insurance purposes. Ethical and legal responsibility for decision making will remain in the hand (better, in the mind) of the natural intelligence of physicians. From this viewpoint, it’s probable that multidisciplinary boards will take the responsibility in difficult cases, considering the information AI provides as relevant but not always conclusive.

## Conclusions

While the application of AI in medicine and radiology has several challenges to face, we should accept the fact that we need it. We need all these technologies and devices that rely on sensitive data to improve patient care and treatment. We believe that challenges such as the new policy initiatives, the regulation of data protection and cybersecurity, the debate about the unusual accountability and responsibility issues, the questions about the fiduciary relationship between patients and AI medical systems will have to be addressed as soon as possible.

AI-based devices could be built to reflect the ethical standards that have guided other actors in healthcare and could be held to those standards. A key step will be determining how to ensure that they are, whether by means of combination of policy enactment and programming approaches.

A good application of AI may be powerful, helpful, and valuable. On the contrary, a bad or unethical use of this cutting-edge technology may be dangerous, and patients, physicians (radiologists), and regulatory authorities must work together to prevent this [57]. In a spirit of good cooperation, we must find a balance that provides security, privacy protection, and ethical use of sensitive information to ensure both humane and regulated (and, therefore, responsible) management of the patients.

## Abbreviations

CT: Computed tomography
EU: European Union
FDA: Food and drug administration
GDPR: General data protection regulation
HIPAA: Health insurance portability and accountability act
IVDR: In vitro diagnostic medical device regulation
MDR: Medical devices regulation
MRI: Magnetic resonance imaging
U.S.: United States

## Footnotes

1
*Data controllers are people who determine the purposes for which and the way any personal data are processed, while a data processor is the person who actually processes the data for a data controller.*
